# 

**Published:** 1916-02-26

**Authors:** 


### WILL EVERY READER PLEASE HELP?

**In the interests of this country, the Empire, and our Allies the Government have intimated an immediate reduction in the importation of paper, paper pulp, and other materials for paper-making. This necessary action of the Government makes it imperative that not a single copy of "The Hospital" shall be wasted, to which end returns must be stopped. This can only be accomplished by readers of "The Hospital" giving an order to their newsagent or bookseller to-day for their weekly copy of "The Hospital." Every reader who at once places an order with his newsagent will co-operate to the full in this matter, and will render the completest possi e help to our country and our Allies.**

### PASSING EVENTS AND TOPICS.

### The City Dispensary.

The Lord Mayor is making an appeal for assistance for the City Dispensary, of which he is President. This charity was founded in 1789, and last year the number of attendances was 15,793. The expenditure was £911, being exactly £1 in excess of the receipts.

### The Dearth of Clinical Thermometers.

One of the minor consequences of the war is a scarcity of clinical thermometers. Consequently the proposal made at a recent meeting of the Leicester Insurance Committee that tuberculosis patients undergoing domiciliary treatment should each be supplied with a thermometer might be difficult to carry out, even if the Committee were willing to agree to a suggestion which appears at first sight to be not without its faults.

### Economy in Prescribing.

The General Purposes Committee of the Panel Committee for the County of London have had under consideration the question of economy in prescribing. They are of opinion that the new system of drug finance is still defective in so far as no provision has yet been made for any differentiation in the amounts of the Drug Funds of the various insurance areas in accordance with the local incidence of sickness; and they recommend that the Insurance Commissioners be asked whether in the event of the sickness incidence in London, or the high cost of drugs, or other causes over which the Committee have no control, being such as to prevent the cost of drugs being kept within the limit of 2s. per insured person, they will be prepared to consider favourably an application from the Committee for a special grant to meet any administrative expenses incurred in checking extravagant prescribing, so that no portion of the expenses so incurred shall fall upon the Practitioners' Fund.

### Hospital Burnt to the Ground.

On Thursday morning last week the main building of the Thetford Small-pox Hospital, which was recently taken over by the military authorities as an isolation hospital, was totally destroyed by fire. Fortunately the thirty patients who were in the building at the time escaped without injury.

### Doctor's Daughter as Locum Tenens.

The Hospital (November 18, 1915, page 140) has already recorded the appointment of a woman doctor as medical officer of health in the place of her husband on active service. The report now comes from Maidstone that the local Board of Guardians has allowed Miss Ethel Shaw, M.R.C.S., L.R.C.P., to act as medical officer during her father's absence on holiday.

### A Festival Luncheon.

The Royal Hospital for Incurables, Putney Heath, announces a festival luncheon in lieu of its annual dinner, to be held on Friday, June 2, at which Sir Frederick Green will preside. The idea of a hospital festival luncheon is a novelty, and it will be interesting to see what it results in during war-time. The dinners of this institution have in recent years produced from £4,000 to £5,000.

---

Sir John Rose Bradford, K.C.M.G., C.B., M.D., F.R.C.P., Colonel A.M.S., has been elected President of the London and Counties Medical Protection Society, Ltd., in place of the late Dr. George Allan Heron.

### Forthcoming Publications.

A New Edition of the St. Thomas's Hospital, Pharmacopœia is in course of preparation by a committee of the medical staff acting with the pharmaceutist, Mr. Jennings.

In the second and enlarged edition of Sir St. Clair Thomson's "Diseases of the Nose and Throat," just published, a description of suspension laryngoscopy has been introduced, and the chapter on the removal of the tonsils has been entirely re-written. The new edition is published by Messrs. Cassell & Co., Ltd.

Sir Alfred Keogh, K.C.B., has written a preface to the new book on "Surgery in War," by Major A. J. Hull, F.R.C.S., R.A.M.C., of which the following is an abstract:—"We are here shown the distinguishing surgical feature of this war, the evolution of the best methods of dealing with the septic processes invariably present, the improvements in immobilising apparatus for gunshot fractures, and many other interesting advances in surgical technique. We get a realising sense of the amount of work which has been done in certain branches of military surgery, such as the surgery of gunshot wounds of the head, of peripheral nerves, and of the blood-vessels. Those, in short, who are familiar with the literature of war surgery will find in almost every chapter matter which is new and suggestive." Several contributors have assisted Major Hull in the compilation of the work. It is well illustrated with *x*-ray plates and diagrams. It will be published by Messrs. J. and A. Churchill.

Pensions for Nurses.—In the matter of the charity hitherto known as "The Institution for Nurses for Nervous and Mental Disorders of Women" the Charity Commissioners have, at the request of the original trustees, promulgated a new scheme for its regulation and administration in substitution for the scheme of 1909-10, which is accordingly repealed. Under the new scheme the title of the charity is to be "The Mrs. Frances Sophia Caldwell Nurses' Fund," and the trustees are to be the corporate body called "The Royal National Pension Fund for Nurses." The operative clauses provide that, after payment of expenses of management, etc., the trustees shall, out of the yearly income, pay an annual pension of £16 13s. 4d. to each of nine persons whose names are specified in a schedule; that, subject to this charge, the income shall be applied in providing pensions at such rates as the trustees may fix, having regard to the needs and circumstances of the pensioners and to the income and other circumstances of the charity. These pensions are to be for nurses who have served for not less than twelve years in connection with cases of nervous and mental disorders of women and are in necessitous circumstances, and each pension will be granted in the first instance for a term of three years, but may be prolonged for a further period of not more than three years at each prolongation. If in any year the whole of the income is not expended, the part remaining unapplied will be invested in augmentation of the capital endowments of the charity. The endowments at present consist of Consols, etc., of the aggregate value of £8,036.—*Lancet.*

Rates of Subscription: Twelve months, 6/6; Six months, 4/-; Three months, 2/-, post free to any address in the United Kingdom. Abroad, Twelve months, 8/8; Six months, 5/-; Three months, 2/6, post free.—Address The Subscription Dept., "The Hospital," The Hospital Building, 28/29 Southampton Street, Strand, London. W.C.

